# Herpes simplex virus type 1 and 2 in mucocutaneous infections

**DOI:** 10.1080/20002297.2017.1325256

**Published:** 2017-06-09

**Authors:** Hannamari Välimaa

**Affiliations:** ^a^ Department of Virology, University of Helsinki, Helsinki, Finland & Department of Oral and Maxillofacial Surgery, Helsinki University Hospital, Helsinki, Finland

## Abstract

Herpes simplex virus (HSV) type 1 and 2 are both known to cause mucosal and skin infections. HSV-1 is regarded as the main virus type in the orofacial region, whereas HSV-2 is mainly associated with genital infections. Recently, an increase in HSV-1 genital infections has been described in some countries.

We investigated the proportion of HSV-1 and HSV-2 isolates from different mucocutaneous regions using diagnostic laboratory database at HUSLAB, Helsinki during the years 2011–2014. Distribution of HSV-1 and HSV-2 among samples was analyzed.

